# Influence of HFE variants and cellular iron on monocyte chemoattractant protein-1

**DOI:** 10.1186/1742-2094-6-6

**Published:** 2009-02-19

**Authors:** Ryan M Mitchell, Sang Y Lee, William T Randazzo, Zachary Simmons, James R Connor

**Affiliations:** 1 George M Leader Family Laboratory, Department of Neurosurgery, Pennsylvania State University College of Medicine/Milton S Hershey Medical Center, Hershey, PA 17033, USA; 2 Department of Neurology, Pennsylvania State University College of Medicine/Milton S Hershey Medical Center, Hershey, PA 17033, USA

## Abstract

**Background:**

Polymorphisms in the *MHC class 1-like *gene known as *HFE *have been proposed as genetic modifiers of neurodegenerative diseases that include neuroinflammation as part of the disease process. Variants of *HFE *are relatively common in the general population and are most commonly associated with iron overload, but can promote subclinical cellular iron loading even in the absence of clinically identified disease. The effects of the variants as well as the resulting cellular iron dyshomeostasis potentially impact a number of disease-associated pathways. We tested the hypothesis that the two most common HFE variants, H63D and C282Y, would affect cellular secretion of cytokines and trophic factors.

**Methods:**

We screened a panel of cytokines and trophic factors using a multiplexed immunoassay in human neuroblastoma SH-SY5Y cells expressing different variants of HFE. The influence of cellular iron secretion on the potent chemokine monocyte chemoattractant protein-1 (MCP-1) was assessed using ferric ammonium citrate and the iron chelator, desferroxamine. Additionally, an antioxidant, Trolox, and an anti-inflammatory, minocycline, were tested for their effects on MCP-1 secretion in the presence of HFE variants.

**Results:**

Expression of the HFE variants altered the labile iron pool in SH-SY5Y cells. Of the panel of cytokines and trophic factors analyzed, only the release of MCP-1 was affected by the HFE variants. We further examined the relationship between iron and MCP-1 and found MCP-1 secretion tightly associated with intracellular iron status. A potential direct effect of HFE is considered because, despite having similar levels of intracellular iron, the association between HFE genotype and MCP-1 expression was different for the H63D and C282Y HFE variants. Moreover, HFE genotype was a factor in the effect of minocycline, a multifaceted antibiotic used in treating a number of neurologic conditions associated with inflammation, on MCP-1 secretion.

**Conclusion:**

Our results demonstrate that HFE polymorphisms influence the synthesis and release of MCP-1. The mechanism of action involves cellular iron status but it appears there could be additional influences such as ER stress. Finally, these data demonstrate a pharmacogenetic effect of HFE polymorphisms on the ability of minocycline to inhibit MCP-1 secretion.

## Background

Iron accumulation in various brain regions is associated with the pathogenesis of several neurodegenerative diseases. The relationship between loss of brain iron homeostasis and the diseases appears to be generation of oxidative stress and exacerbation of inflammatory responses. The degree to which loss of iron homeostasis contributes to cell dysfunction and loss is not clear. Cellular iron homeostasis involves the complex interaction of numerous proteins, and genetic variants in genes encoding these proteins may influence this process and hence pathogenesis and disease progression. Polymorphisms in the *major histocompatibility complex class 1-like *gene known as *HFE *have been proposed as genetic modifiers of amyotrophic lateral sclerosis (ALS) [1], Alzheimer's disease (AD) [2], Parkinson's disease (PD) [3], and stroke [4]. The HFE protein is primarily thought to regulate transferrin-mediated cellular iron intake through its interaction with the transferrin receptor [5], but a recent study from our laboratory suggests a potentially broader role in cell function [6]. The H63D HFE variant appears deficient in reducing the affinity of the transferrin receptor for transferrin, while the C282Y HFE variant aggregates in the endoplasmic reticulum [5,7]. These HFE variants are thought to underlie the iron overload disorder known as hemochromatosis, but appear to have low penetration rates [8]. Nonetheless, there is little disagreement that HFE variants impact iron status at the cellular level independent of clinical disease [5-7,9].

Cellular iron dysregulation has the potential to indirectly impact numerous disease-associated pathways. Our study is focused on the link between cellular iron regulation and secretion of soluble mediators of inflammatory reactions [10,11]. Iron dysregulation in neuronal cells induces toxicity [12], potentially resulting in recruitment of inflammatory cells [13]. One factor proposed to contribute to the relentless progression associated with many neurodegenerative diseases is the recruitment and activation of microglia in the regions of neuronal death [14]. As mediators of innate immunity in the central nervous system, microglia respond to a variety of cytokines and chemokines and in turn secrete a variety of inflammatory mediators and trophic factors which likely contribute to neurodegenerative disease pathogenesis.

By influencing cellular iron homeostasis, we hypothesize that HFE variants may differentially impact secretion of cytokines and trophic factors, which may explain the association of these alleles with various diseases. In the current study we determine the influence of HFE variants and iron regulation on cellular secretion of factors relevant to neurodegenerative disease and explore mechanisms underlying these relationships.

## Methods

### Materials

SH-SY5Y human neuroblastoma cell lines and human astrocytoma cell lines U251 and U138 MG, were obtained from American Type Culture Collection (Manassas, VA). Mouse microglial BV-2 cells were a kind gift of Dr. Steven W. Levison (University of Medicine & Dentistry of New Jersey). Cell culture reagents including DMEM/F12, DMEM, penicillin/streptomycin, penicillin/streptomycin/glutamine and Geneticin, as well as calcein-AM were purchased from Invitrogen (Carlsbad, CA). Fetal bovine serum was purchased from Gemini Bio-Products (West Sacramento, CA). MTS reagent was purchased from Promega (Chatsworth, CA). PCR primers for MCP-1 were purchased from SuperArray (Frederick, MD). The DNeasy Tissue kit and RNeasy Mini kit were purchased from Qiagen (Valencia, CA). The DC protein assay and the Bio-Plex Human 27-plex panel of cytokines and growth factors were obtained from Bio-Rad (Hercules, CA). Anti-Hsp70 and anti-Hsp90 antibodies were purchased from Stressgen (Ann Arbor, MI). All other reagents were purchased from Sigma-Aldrich (St. Louis, MO).

### Cell culture

**Figure 1:**
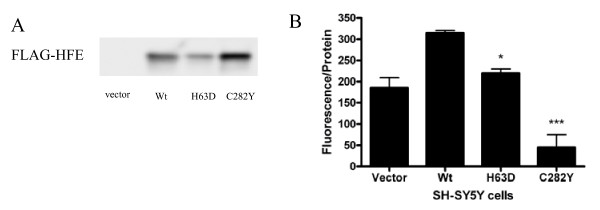
**Confirmation of cell transfection**. (A) A representative western blot is shown demonstrating FLAG-tagged HFE expression in transfected SH-SY5Y cells (B) Intracellular labile iron was determined in the differentiated SH-SY5Y cells using the cell-permeable dye calcein-AM, as described in Methods. Fluorescence of calcein is quenched in the presence of iron, thus fluorescence is inversely proportional to labile iron levels. Intracellular labile iron levels were in the order C282Y > H63D = Vector > Wt. Results are reported here as the average ± SEM of three independent assays. * and *** indicate p < 0.05 and p < 0.001, respectively, compared to Wt.

As previously reported [1], we created stably-transfected human neuroblastoma SH-SY5Y cell lines expressing FLAG-tagged wildtype (Wt), H63D, and C282Y forms of HFE. This cell line was chosen because it lacks endogenous HFE expression [1,6]. As a control, we also transfected cells with vector alone. Transfected cells were maintained in DMEM/F12 medium supplemented with 10% FBS, 1% penicillin/streptomycin/glutamine, 1× nonessential amino acids, 1.8 g/L sodium bicarbonate, and 250 μg/mL Geneticin. Prior to use, cells were differentiated for four to six days in 10 μM all-trans retinoic acid and the genotype of each cell line was confirmed by sequencing. Vector control cells were included in analyses to control for phenotypic changes associated with the cell transfection. The more direct control for the HFE polymorphisms, however, is the Wt HFE transfected cells, because of the effect of Wt/Wt HFE on cellular iron status that does not occur in the cells transfected with vector alone which do not express HFE (Figure 1).

Astrocytoma cell lines were maintained in Dulbecco's Modified Eagle's Medium supplemented with 10% fetal bovine serum and 1% penicillin/streptomycin. BV-2 cells were maintained in DMEM supplemented with 10% heat-inactivated FBS and 1% penicillin/streptomycin. Cells were generally used after seven to ten (maximum) passages.

### HFE genotyping

Genomic DNA was extracted from astrocytoma cell lines using the DNeasy Tissue kit according to the manufacturer's instructions. The PCR-amplified DNA samples were digested by restriction enzymes to determine the *HFE *genotype [15]. DNA (50 ng) was amplified by PCR using the primers forward: 5'-ACA TGG TTA AGG CCT GTT GC-3', and reverse: 5' CTT GCT GTG GTT GTG ATT TTC C-3' for detection of the *H63D *allele, and primers forward: 5' CAA GTG CCT CCT TTG GTG AAG GTG ACA CAT-3', and reverse: 5' CTC AGG CAC TCC TCT CAA CC-3' for the *C282Y *allele. The PCR reaction was performed in a total volume of 50 μL containing 100 pmol of each primer in a PTC-200 Peltier Thermal Cycler (MJ Research, Woburn, MA). The PCR reaction was initiated at 95°C for 15 min, followed by 40 cycles of denaturation at 95°C for 45 s, annealing at 58°C for 45 s, and extension at 72°C for 1 min 30 s. A 294 bp product was obtained for the *H63D *polymorphism. Following digestion with *MboI*, the *63D *allele gave restriction fragments of 237 and 57 bp, while the *H63 *allele resulted in three fragments (138, 99 and 57 bp). For the *C282Y *polymorphism, the primers resulted in a 343 bp product. Following digestion with *RsaI*, the *282Y *allele resulted in products of 203, 111 and 29 bp, whereas the *C282 *allele yielded restriction fragments of 203 and 140 bp. Polymorphisms were detected by restriction fragment length analysis in 5% TBE polyacrylamide gel.

### Cell viability assays

Differentiated SH-SY5Y cell lines were grown in 96-well plates until 70–80% confluent. Cells were treated with various concentrations of desferroxamine (DFO), ferric ammonium citrate (FAC), Trolox and minocycline. Cell viability was assessed with the colorimetric agent MTS and compared to untreated cells.

### Western blotting

SH-SY5Y cells were grown as separate cultures until approximately 80% confluent. Cells were lysed with a RIPA buffer supplemented with 1% Triton X-100 and protease inhibitor cocktail. Forty micrograms total protein was separated by electrophoresis in a 4–20% Criterion polyacrylamide gel. Protein was then transferred to a nitrocellulose membrane and blocked for 1 hr at room temperature in TBS-T with 5% nonfat milk. Membranes were then probed with anti-FLAG (1:2000), anti-Hsp70 (1:1000) and anti-Hsp90 (1:1000) primary antibodies in TBS-T with 5% nonfat milk overnight at 4°C. HRP-conjugated secondary antibodies (1:5000) were then added in 5% nonfat milk for 1 hr at room temperature. Signals were obtained by chemiluminescence and visualized by CCD camera. Each blot shows results obtained from three independent cell cultures.

### Intracellular labile iron

Intracellular labile iron was determined in the SH-SY5Y cells using the cell-permeable dye calcein-AM. Cells were grown in 24-well plates to 80% confluency. Calcein-AM was then added in medium at a final concentration of 0.25 μM and cells were incubated at 37°C for 30 minutes. Cells were then washed three times in ice-cold Hanks' balanced salt solution and lysed in 200 μL RIPA buffer plus 1% Triton X-100. The fluorescence of the lysate was measured and normalized to the protein content of the lysate, determined by BioRad DC protein assay, after subtracting a blank consisting of cells incubated with medium lacking the dye.

### Multiplex cytokine bead assay

We performed multiplex analysis on undiluted cell culture supernatants using the Bio-Plex Human 27-plex panel of cytokines and growth factors listed in Table 1 (Bio-Rad; Hercules, CA). Briefly, cells were plated in 24-well plates and grown to approximately 70% confluency. Medium was then changed, and cells were incubated for 48 hrs. Cells were lysed in RIPA buffer after samples of media were removed and analyzed by a multiplex antibody-based assay. Fifty μL of each sample or standard was added in duplicate to a 96-well filter plate and mixed with 50 μL of antibody-conjugated beads for one hour at room temperature. After one hour, wells were washed and 25 μL of detection antibody was added to each well. After 30 minutes incubation, wells were washed and 50 μL of streptavidin-PE was added to each well and incubated for 10 minutes. A final wash cycle was then completed and 125 μl of assay buffer was added to each well. The plate was then analyzed using a Bio-Plex 200 workstation (Bio-Rad). Analyte concentration was calculated based on the respective standard curve for each cytokine.

**Table 1:** Cytokines and trophic factors assayed with bead-based immunoassay (Bio-Rad; Hercules, CA)

Eotaxin	IL-4	IL-15
fibroblast growth factor (FGF)	IL-5	IL-17
granulocyte colony stimulating factor (G-CSF)	IL-6	monocyte chemoattractant protein-1 (MCP-1)
granulocyte-macrophage colony stimulating factor (GM-CSF)	IL-7	macrophage inflammatory protein-1α (MIP-1α)
interferon-gamma	IL-8	MIP-1β
IFN-γ induced protein-10 (IP-10)	IL-9	platelet derived growth factor (PDGF) bb
interleukin-1beta (IL-1β)	IL-10	regulated on activation normal T-cell expressed and presumably secreted (RANTES)
IL-1 receptor antagonist (IL-1ra)	IL-12(p70)	tumor necrosis factor-alpha (TNF-α)
IL-2	IL-13	vascular endothelial growth factor (VEGF)

### Measurement of MCP-1 release

Cells were plated in 24-well plates and grown to ~70% confluency. The medium was replaced and cells were incubated for 48 hrs, after which time medium was collected, cells were washed two times in Hanks' balanced salt solution, and cells were lysed in RIPA buffer plus 1% Triton X-100. Protein content of the cell lysate was determined by DC protein assay. MCP-1 concentrations in the conditioned medium were determined using commercially available anti-human (SH-SY5Y or astrocytoma cells) or anti-mouse (BV-2 cells) MCP-1 ELISA (GE Healthcare, Piscataway, NJ), and reported as MCP-1 normalized to the protein content of the cell lysate. The impact of iron loading and iron deprivation on MCP-1 release was determined in each cell line. Each cell line was treated with varying concentrations of ferric ammonium citrate (FAC) or desferroxamine (DFO) at levels below toxic concentrations, determined by MTS cytotoxicity assay. Additionally, MCP-1 release was determined in transfected SH-SY5Y cells treated with 50 or 100 μM Trolox or 25 μM minocycline, all concentrations determined to be below toxic levels. For FAC, DFO, Trolox, or minocycline treatment, agents were added to cell culture medium when medium was changed after cells reached ~70% confluency, resulting in treatment for 48 hours.

### Quantitative reverse-transcription polymerase chain reaction (qRT-PCR)

We performed qRT-PCR for MCP-1 and GAPDH mRNA expression in SH-SY5Y cells using PCR primers commercially available from SuperArray. Total RNA was extracted from five independent cell cultures using the RNeasy Mini kit (Qiagen) and 5 μg of total RNA was used in a 20 μL cDNA reaction mixture using the ReactionReady First Strand cDNA synthesis kit (SuperArray). PCR was performed on cDNA using the RT^2 ^Real-Time SYBR Green assay (SuperArray) with an ABI 7300 (Applied Biosystems, Foster City, CA) real-time PCR system. The reaction was initiated at 95°C for 10 min, followed by 40 cycles of denaturation at 95°C for 15 s, and annealing and extension at 60°C for 1 min. Expression levels of MCP-1 were normalized to expression of GAPDH mRNA.

### NF-κB

Nuclear fractions were isolated from four independent cultures of each stably-transfected SH-SY5Y cell line using a nuclear extraction kit (Millipore, Billerica, MA). Nuclear extracts were analyzed for NF-κB (p50) content by commercially-available colorimetric assay (Cayman Chemical, Ann Arbor, MI), and results are expressed as optical density per milligram of nuclear protein.

### Statistical analysis

Data are shown as mean ± SEM., with each n representing an independent cell culture. The data were analyzed by one-way ANOVA with Tukey's post-hoc analysis or Student's *t *test, as appropriate. Differences among means were considered statistically significant when the p-value was < 0.05.

## Results

Stably-transfected SH-SY5Y human neuroblastoma cells expressing different variants of HFE were generated as previously reported [1]. Prior to use, cells were differentiated with 10 μM all-trans retinoic acid to induce a neuronal phenotype. Expression of FLAG-tagged HFE was confirmed in the cells, as shown in Figure 1A. Relative iron levels were determined in cells using the iron sensitive dye calcein-AM, whose fluorescence is quenched by binding iron. The cells transfected with vector alone do not contain HFE and have iron levels similar to the cells carrying the H63D alleles. Intracellular iron is decreased by the presence of the Wt HFE, consistent with the known functions of the HFE protein, compared to the vector alone group as well as the cells carrying the HFE allelic variants. To determine the effects of HFE polymorphisms, the comparisons are limited to the cells expressing HFE and thus do not include the vector alone transfected cells. The intracellular level of labile iron was affected by the presence of HFE in these cells in the pattern of C282Y (p < 0.001 vs. Wt) > H63D (p < 0.05 vs. Wt) > Wt (Figure 1B). These data demonstrate that the cells in our study had differences in the labile iron pool associated with the different polymorphisms and are generally consistent with our previous report [6].

**Figure 2:**
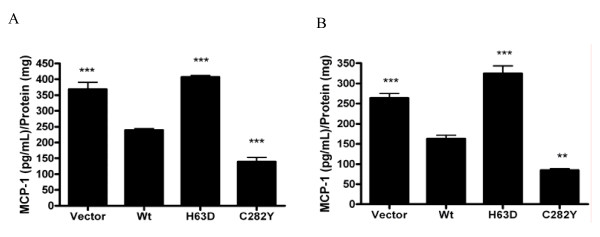
**MCP-1 secretion in SH-SY5Y cells**. (A) MCP-1 concentrations were measured in conditioned medium from differentiated SH-SY5Y cells and normalized to the protein content of the cell lysis solution and reported here as the average ± SEM of five independent assays. Cells expressing H63D HFE released 71% more MCP-1 than cells expressing Wt HFE, while cells expressing C282Y HFE released 42% less MCP-1 than Wt-expressing cells. C282Y cells released significantly less MCP-1 than all other cell lines. (B) Similar results were obtained using undifferentiated cells. **, and *** represent p < 0.01, and p < 0.001, respectively, compared to Wt HFE cells.

The effects of HFE variants on the release of soluble mediators were determined using the transfected SH-SY5Y cells. A multiplexed antibody-based bead assay was used to screen a prospective panel of analytes consisting of inflammatory and anti-inflammatory cytokines and trophic factors. Eotaxin, IFN-γ, IL-1 receptor antagonist, IL-7, IL-9, IL-12(p70), IL-13, MCP-1, RANTES, and VEGF were measured in detectable quantities in cell-conditioned medium from vector-transfected cells or cells expressing Wt and/or H63D HFE. Of these factors, only the difference in MCP-1 reached statistical significance between cells expressing Wt HFE and those expressing H63D HFE (p < 0.001). Over 48 hours, cells expressing H63D HFE released 71% more MCP-1 than cells expressing Wt HFE (p < 0.001), while cells expressing C282Y released 42% less MCP-1 than Wt-expressing cells (p < 0.001), as shown in Figure 2A. Similar results were also obtained using undifferentiated cells (Figure 2B). The expression of MCP-1 in the H63D and vector-only cells, like the levels of intracellular iron, was similar.

**Figure 3:**
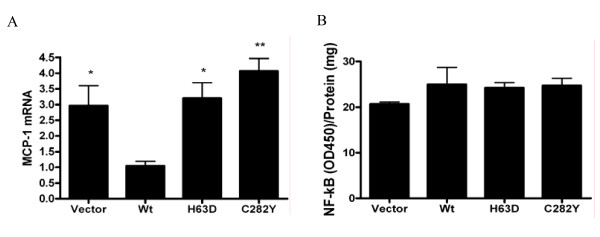
**MCP-1 mRNA expression and nuclear NF-κB**. (A) RNA was isolated from five separate cultures of differentiated SH-SY5Y cells and used for quantitative RT-PCR. Expression of MCP-1 mRNA was normalized to the expression of GAPDH mRNA. MCP-1 mRNA expression was 3.0-, 3.2-, and 4.1-fold higher in vector-transfected, H63D HFE, and C282Y HFE cells, respectively, compared to cells expressing Wt HFE. (B) Nuclear fractions were isolated from four independent cultures of stably-transfected SH-SY5Y cell lines. Nuclear content of NF-κB (p50) was assessed by colorimetric assay and reported as optical density per milligram of nuclear protein. Results are expressed as mean ± SEM. No differences in NF-κB (p50) were found between any of the SH-SY5Y cell lines. n = 4. * and ** represent p < 0.05 and p < 0.01, respectively, compared to Wt HFE cells.

The mechanism by which HFE variants may differentially regulate synthesis of MCP-1 was explored at the transcription level. RNA was isolated from each SH-SY5Y cell line for measurement of MCP-1 mRNA expression. After normalizing to the expression of GAPDH mRNA, MCP-1 mRNA expression was 3.0- (p < 0.05), 3.2- (p < 0.05), and 4.1-fold higher (p < 0.01) in vector-transfected, H63D HFE, and C282Y HFE cells, respectively, compared to cells expressing Wt HFE (Figure 3A). Between cells expressing Wt HFE and those expressing H63D HFE or even the vector alone cells, MCP-1 mRNA expression followed the pattern of MCP-1 secretion. Despite less secretion of MCP-1 associated with the C282Y HFE variant compared to H63D HFE, however, MCP-1 mRNA expression was not significantly different between the two cell lines. These data suggest MCP-1 is regulated at both the transcriptional and post-transcriptional levels and that the mutant proteins or iron status can affect the regulation.

NF-κB is known to have a prominent role in the transcriptional regulation of many inflammatory mediators including MCP-1 [16]. Analysis of the nuclear content of NF-κB in the SH-SY5Y cell lines, however, revealed no differences associated with any of the HFE variants (Figure 3B).

**Figure 4:**
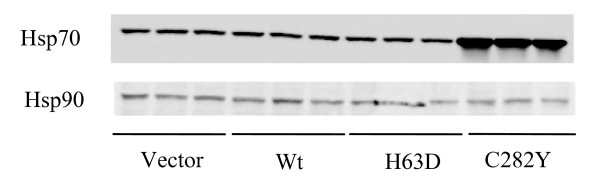
**Heat shock protein expression**. Expression of Hsp70 and Hsp90 were determined in differentiated SH-SY5Y cell lines by western blotting, demonstrating increased expression of Hsp70 in C282Y HFE cells. Samples from three separate cell cultures for each cell line were analyzed on the same gel.

Heat stress, likely mediated through induction of heat shock proteins, reportedly decreases production of MCP-1 [17]. SH-SY5Y cells were assessed for expression of the heat shock proteins Hsp70 and Hsp90. Cells expressing C282Y HFE were characterized by increased expression of Hsp70 without an increase in Hsp90 expression. Other cell lines showed no differences in expression of either protein (Figure 4). H63D HFE and C282Y HFE cells showed similar levels of MCP-1 mRNA, but significantly different levels of secreted protein, suggesting that the elevated Hsp70 levels in the C282Y HFE cells may be affecting post-transcriptional or post-translational regulation of MCP-1.

**Figure 5:**
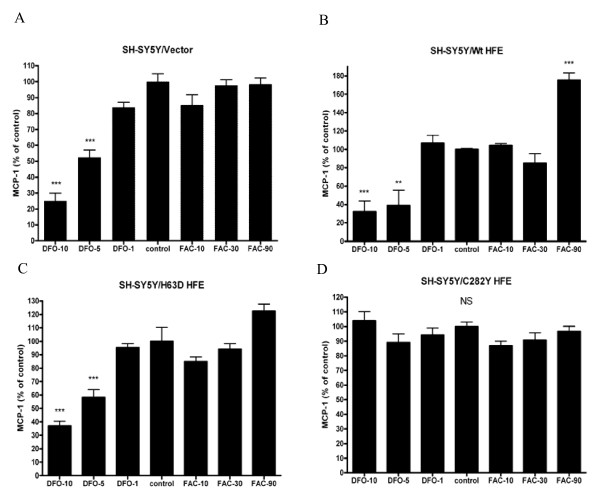
**Impact of iron on MCP-1 secretion**. Vector-transfected SH-SY5Y cells (A) and SH-SY5Y cells expressing Wt HFE (B), H63D HFE (C), or C282Y HFE (D) were treated with 10 μM, 5 μM, or 1 μM desferroxamine (DFO) or 10 μM, 30 μM, or 90 μM ferric ammonium citrate (FAC) for 48 hours and the secretion of MCP-1 was determined by ELISA and normalized to cellular protein content. n = 4. *, **, and *** indicate p < 0.05, 0.01, and 0.001 compared to control. NS = not significant.

To directly determine the ability of iron to influence MCP-1 secretion from cells, each cell line was treated with non-toxic doses of DFO and FAC to interrogate the effect of cellular iron modulation on MCP-1 release (Figure 5). DFO treatment resulted in dose-dependent decreased MCP-1 release in vector-transfected (maximum 75%, p < 0.001), Wt HFE (maximum 68%, p < 0.001), and H63D HFE cells (maximum 64%, p < 0.001), at 5 μM and 10 μM, but had no effect in C282Y HFE cells at these concentrations. Treatment with 90 μM FAC resulted in significantly more MCP-1 secretion in Wt HFE cells (maximum 76%, p < 0.001), but had no effect at any concentration in other cell lines. Treatment with either DFO or FAC had no significant effects in cells expressing C282Y HFE, at these concentrations. Higher concentrations of DFO and FAC were not examined because of data suggesting toxicity at higher doses of these two agents (data not shown).

**Figure 6:**
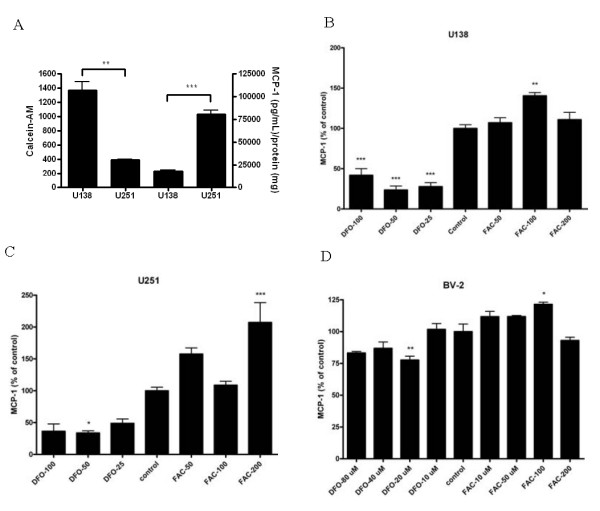
**MCP-1 secretion in astrocytoma and microglial cells**. Secretion of MCP-1 in U138 (*H63D/Wt*) and U251 (*Wt/Wt*) human astrocytoma cell lines and mouse microglial BV-2 cells was determined with various concentrations of DFO or FAC, as reported in Methods. MCP-1 concentration in medium was determined, relative to the protein content of each well, and was normalized to control levels. n = 4. *, **, and *** represent p < 0.05, p < 0.01, and p < 0.001, respectively, compared to control.

In addition to neurons, within the CNS astrocytes and microglia are also sources of MCP-1 secretion [13]. Therefore, as an additional model, we utilized two human astrocytoma cell lines, U251 and U138, to study the effect of cellular iron status on MCP-1 secretion. Both cell lines were genotyped for the *H63D *and *C282Y HFE *variants (S.Y. Lee, unpublished). The *Wt/Wt HFE *U251 cells were characterized by higher intracellular labile iron levels (p < 0.01) compared to the *H63D/Wt HFE *U138 cells and had higher constitutive MCP-1 release (p < 0.001) (Figure 6A). In order to determine if iron levels were related to the differences in MCP-1 secretion, cells were treated with DFO or FAC. Following treatment with DFO (50 μM), the secretion of MCP-1 was reduced by 77% (p < 0.001) in U138 cells (Figure 6B) and by 66% (p < 0.05) in U251 cells (Figure 6C). Iron loading (100 μM FAC) resulted in a 41% increase of MCP-1 secretion in U138 cells (p < 0.01) with, but this concentration of FAC had no effect in U251 cells. A doubling of the FAC concentration to 200 μM did increase MCP-1 secretion by 107% in U251 cells (p < 0.001). Thus, regardless of endogenous *HFE *genotype in the astrocytoma cell lines, secretion of MCP-1 was reduced by similar concentrations of an iron chelator, and MCP-1 secretion could be induced by iron; the effective concentration of iron did differ based on HFE genotype. Additionally, the influence of iron on MCP-1 secretion was studied in mouse microglial BV-2 cells (Figure 6D). Iron chelation with 20 μM DFO produced a maximal inhibition of MCP-1 release of 22% (p < 0.01), and treatment with 100 μM FAC produced a maximal increase in MCP-1 release of 22% (p < 0.05). In both astrocytoma cell models and the BV-2 cell model, increasing or decreasing cellular iron was directly correlated with MCP-1 release.

**Figure 7:**
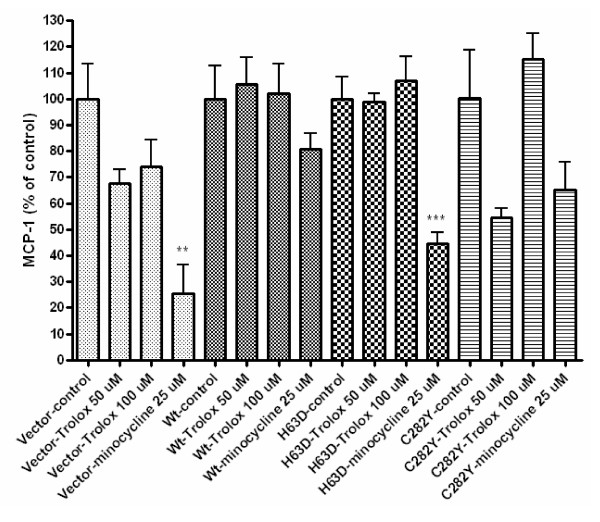
**Trolox and minocycline effects on MCP-1 secretion**. Stably transfected SH-SY5Y cells were treated for 72 hours with Trolox or 48 hours with minocycline to determine the impact of these agents on MCP-1 release. Trolox at either concentration had no significant effect of MCP-1 release in any of the SH-SY5Y cell lines in this study. Minocycline treatment resulted in a 76% decrease (p < 0.01) and 56% decrease (p < 0.001) in MCP-1 release from vector and H63D cells, respectively, but no significant effects in Wt or C282Y cells. n = 4. **, and *** indicate p < 0.01, and p < 0.001, respectively, compared to baseline MCP-1 secretion for each respective cell line.

The iron chelation and iron addition experiments indicate that cellular iron status can influence MCP-1 secretion. Iron status also affects the level of cellular oxidative stress. Therefore to investigate the possibility that oxidative stress could be influencing the release of MCP-1, the stably transfected SH-SY5Y cells were treated for 48 hours with Trolox, a water-soluble form of vitamin E. Additionally, cells were treated with minocycline, an antibiotic with iron chelation, antioxidant, anti-inflammatory, and mitochondria-protective properties [18-21], because this drug has been explored for the treatment of neurodegenerative diseases. These doses and duration of treatment were chosen based on cell viability assays (data not shown). Trolox at either concentration had no significant effect on MCP-1 release in any of the SH-SY5Y cell lines in this study. Minocycline treatment resulted in a 76% decrease (p < 0.01) and 56% decrease (p < 0.001) in MCP-1 release in vector-transfected and H63D HFE cells, respectively, but no significant effects in cells expressing Wt or C282Y HFE (Figure 7).

## Discussion

The analysis of the panel of cytokines and trophic factors revealed that MCP1 is the most sensitive to cellular iron status and HFE expression. MCP-1 is known to recruit and enhance the activation of cells of the monocyte lineage [22]. Higher levels of MCP-1 have been found in CSF and plasma in association with diseases characterized by excessive inflammation including ALS and AD [23,24] and in both of these diseases multiple reports exist that *HFE *gene variants, particularly the *H63D *polymorphism, are risk factors for the disease [1,2,25]. Additionally, previous studies from our group and others [23,26] have determined a positive correlation between age and expression of MCP-1, which may play a role in the higher incidence of neurodegenerative diseases at advanced ages. Furthermore, Lawless et al. [27] demonstrated that individuals with hemochromatosis who were homozygous for the *C282Y *allele had lower median plasma levels of MCP-1. Those hemochromatosis patients with at least one *H63D *allele, however, had higher median plasma levels of MCP-1 compared to *Wt/Wt *control subjects. These findings are consistent with the data using neuroblastoma cells reported herein.

In this study we have utilized a stably-transfected human neuroblastoma cell culture model to elucidate the consequences of *HFE *gene variants on MCP-1 expression. There are two key observations in this study. First is that H63D-expressing neuroblastoma cells have more iron than Wt HFE cells and secrete more MCP1. Secondly, the relationship between MCP1 secretion and iron is altered in the presence of the C282Y HFE variant via a mechanism which appears to be post-transcriptional or post-translational. The decreased expression of MCP-1 in the C282Y HFE cells may be related to the increased expression of Hsp70 in these cells. Our results on MCP-1 secretion in the C282Y cells are not consistent with a report using transiently-transfected HEK-293 cells [28]. Our data suggest that iron could be a driving factor behind the secretion of MCP-1 and the transient transfection of cells already expressing Wt HFE may not compare well to our model. The expression of both Wt and C282Y forms of HFE in a cell model may have a confounding effect on MCP-1 secretion. It is also possible that the effect on MCP-1 secretion, as shown with the non-transfected astrocytoma cells in our study, is cell specific.

To determine the effect of cellular iron status on MCP-1 secretion, the cells were treated with either an iron chelator or iron was added to the media. In both the neuroblastoma cells and astrocytoma cells, the Wt and H63D cells and the non-HFE expressing vector control neuroblastoma cells, chelating the iron with DFO resulted in a decrease in MCP-1 expression. Iron chelation failed to decrease MCP-1 expression in the C282Y HFE cells. The addition of iron increased MCP-1 expression only in Wt neuroblastoma cells and both genotypes in the astrocytoma cells. Thus in general, it appears that the expression of MCP-1 can be manipulated by altering iron status. There is, however, an interaction of genotype and cell type that influences the MCP-1 expression independent of iron status. For example, the Wt neuroblastoma cells have less iron and less MCP-1 expression than the H63D-expressing cells. However, the reverse occurs in the astrocytoma cell lines; the Wt cells have more iron in the labile iron pool and more MCP-1 expression than the cells carrying the H63D allele. The astrocytoma cells are heterozygotic for the H63D variant and thus the baseline differences in iron status from Wt/Wt and the effect on MCP-1 secretion may reflect a heterozygotic effect. The astrocytoma cells also differ in genetic background. In the context of our study, regardless of cell type and *HFE *genotype, MCP-1 secretion was increased by increasing cellular iron and decreased by iron chelation, with the exception of cells expressing C282Y HFE. Thus it appears that C282Y HFE has an independent effect on secretion of MCP-1 that negates the effects of iron.

These data suggest that there is an additional function of the C282Y HFE mutant protein that is impacting MCP-1 secretion. Effects of the C282Y HFE variant contributing to the iron overload condition, hereditary hemochromatosis, have been suggested to result from induction of the ER overload and unfolded protein responses [28]. Subsequent effects of these latter responses include activation of NF-κB [29] and potential activation of apoptosis [30], which have been associated with overexpression of C282Y HFE in one cell model [28]. However, we found no difference in nuclear localization of NF-κB in our cell lines and have suggested a post-transcriptional or post-translational effect on MCP-1 that may be directly influenced by the mutant HFE protein.

The relationship between iron status, HFE status, and MCP-1 secretion prompted analysis of the known regulatory mechanisms for MCP-1 in these cells. MCP-1 is thought to be regulated mainly at the transcriptional level, responding to a variety of transcription factors. In neuroblastoma cell lines, the presence of the H63D and C282Y forms of HFE were associated with higher levels of MCP-1 mRNA than in the wildtype cells. The levels of MCP-1 mRNA were similar to that seen in the vector only cells. A large number of studies have explored the upregulation of MCP-1 by NF-kB, an oxidative stress-sensitive transcription factor which might explain the relationship between increased iron and MCP-1. However, nuclear localization of NF-κB did not vary by *HFE *genotypes, and thus iron status, in our model, consistent with a previous report that iron status did not affect baseline nuclear NF-κB in microglial cells [10].

A likely mechanism through which cellular iron status affects cell stress and MCP-1 regulation is through the formation of reactive oxygen species. In the present study the antioxidant Trolox had no significant effect on MCP-1 secretion in any of the SH-SY5Y cell lines. It is possible that the length of treatment (72 hours) was not sufficient to impact MCP-1 synthesis or release; however, longer duration of treatment showed evidence of cellular toxicity (data not shown).

An additional pharmaceutical intervention we examined was minocycline. Minocycline has been explored for use in treating a number of neuroinflammatory conditions including ALS, AD, PD, multiple sclerosis, and ischemic stroke. This compound has iron chelation [18], antioxidant [19], anti-inflammatory [31], caspase-inhibitory [31], and mitochondria-protective properties [32]. In the present study, effects of treating cells with minocycline varied with *HFE *genotype, significantly decreasing MCP-1 release in the two SH-SY5Y cell lines with the highest MCP-1 secretion.

The multifaceted nature of minocycline causes uncertainty of the specific contribution of each mechanism of action. Minocycline, like other tetracyclines, has iron chelation properties [18]. The ability to reduce intracellular iron levels may explain the benefit of minocyline in reducing MCP-1 secretion in the relatively iron-laden H63D HFE cells. The lack of effect seen in Wt HFE cells, which responded to iron chelation with DFO, may result from other effects of minocycline, or simply reflect the relative differences in chelator strengths between DFO and minocycline. An alternative explanation for the differential effects of minocycline involves its actions on mitochondria. Minocycline has been shown to have mitochondrial protective properties, including reduction of mitochondrial swelling and permeability transition pore opening [32]. Mitochondrial dysfunction in the presence of HFE gene variants has been suggested for both the C282Y allelic variant [28] and the H63D HFE variant [6]. Additionally, we have determined that the H63D HFE variant is associated with elevated cytosolic calcium levels (manuscript under review), that may result from altered mitochondrial function. Mitochondrial dysfunction and elevated calcium levels are mechanisms proposed to induce transcription of MCP-1; however, the association of mitochondrial function with MCP-1 regulation is mostly regulated through NF-κB [33]. Thus, in contrast to its other actions, the data herein suggest that the effects of minocycline on MCP-1 secretion involve iron chelation. Although the exact mechanism of minocycline is not identified in these cells, these data strongly suggest that HFE genotype should be considered when assessing treatment efficacy of minocycline, and perhaps other pharmacological agents. The lack of benefit of minocycline in the treatment of humans with ALS, for example [31], despite its benefit in the mouse model [33], may be due to patient heterogeneity for HFE alleles. Additional studies of minocycline in patients with ALS may be warranted if they include stratification by *HFE *genotype because approximately 30% of ALS patients carry at least one *H63D or C282Y HFE *allele [1,25]. Given the broad effects of HFE on regulation of MCP-1, such stratification may be justified in other clinical trials as well.

## Abbreviations

AD: Alzheimer's disease; ALS: amyotrophic lateral sclerosis; ANOVA: analysis of variance; β2M: beta 2 microglobulin; CNS: central nervous system; CSF: cerebrospinal fluid; DFO: desferroxamine; DMEM: Dulbecco's modified Eagles Medium; ELISA: enzyme-linked immunosorbent assay; FAC: ferric ammonium citrate; HIF: hypoxia inducible factor; HRP: horseradish peroxidase; Hsp: heat shock protein; MCP-1: monocyte chemoattractant protein-1; MHC: major histocompatibility complex; MTS: 3-(4,5-dimethylthiazol-2-yl)-5-(3-carboxymethoxyphenyl)-2-(4-sulfophenyl)-2H-tetrazolium; NF-κB: nuclear factor-kappa B; PD: Parkinson's disease; qRT-PCR: quantitative reverse-transcription polymerase chain reaction; SEM: standard error of the mean; TBS-T: Tris-buffered saline-Tween; Wt: wildtype.

## Competing interests

The authors declare that they have no competing interests.

## Authors' contributions

RMM, WTR, ZS and JRC designed the experiments. SYL performed cell transfection. RMM and WTR performed all other experiments. ZS and JRC directed this work, and contributed to final data analysis. RMM and JRC wrote the manuscript. All authors assisted with, edited and approved the final manuscript.
